# Notoginsenoside R1 alleviates spinal cord injury through the miR-301a/KLF7 axis to activate Wnt/β-catenin pathway

**DOI:** 10.1515/med-2022-0461

**Published:** 2022-04-13

**Authors:** Zhi Tang, Chunhua Yang, Zhengwen He, Zhiyong Deng, Xiaoming Li

**Affiliations:** Department of Neurosurgery, Hunan Cancer Hospital, The Affiliated Cancer Hospital of Xiangya School of Medicine, Central South University, Changsha 410013, Hunan, China; Department of Orthopaedics, The First Hospital of Changsha, Kaifu District, Changsha 410005, Hunan, China; Department of Orthopaedics, The First Hospital of Changsha, No. 311, Yingpan Road, Kaifu District, Changsha 410005, Hunan, China

**Keywords:** notoginsenoside R1, miR-301a, KLF7, spinal cord injury

## Abstract

Spinal cord injury (SCI) is a devastating incident that induces neuronal loss and dysfunction. Notoginsenoside R1 (NGR1) has been reported to exhibit a neuroprotective role after SCI. In this study, the effect and molecular mechanisms of NGR1 in models of SCI were further investigated. Rat adrenal pheochromocytoma cell line (PC-12) were stimulated with lipopolysaccharide (LPS) to establish a cell model of SCI-like condition. The changes of proinflammatory cytokines and associated proteins were analyzed using enzyme linked immunosorbent assay (ELISA) and western blotting. A rat model of SCI was established. Nissl staining were used to observe the morphological characteristics of spinal cord tissues. reverse transcription-quantitative PCR (RT-qPCR) was used to measure the expression of miR-301a andKrüppel-like factor 7 (KLF7). Our results showed that NGR1 alleviated LPS-triggered apoptosis and inflammation in PC-12 cells. MiR-301a was upregulated in LPS-stimulated PC-12 cells and was downregulated by NGR1 treatment. MiR-301a overexpression reversed the effect of NGR1 in LPS-treated PC-12 cells. KLF7 was verified to be targeted by miR-301a. NGR1 activated Wnt/β-catenin signaling in LPS-treated PC-12 cells by inhibiting miR-301a and upregulating KLF7. Moreover, blocking wingless/integrated (Wnt)/β-catenin signaling eliminated the protective effect of NGR1 against SCI *in vitro* and *in vivo*. Overall, NGR1 could reduce inflammation and apoptosis and promote functional recovery of SCI rats by activating Wnt/β-catenin pathway.

## 1 Introduction

Spinal cord injury (SCI) is a serious neurological injury in the central nervous system caused by fracture or dislocation of the spine [1]. The pathological process of SCI is typically divided into two phases: primary and secondary injury. Secondary SCI is mainly promoted by inflammatory response caused by the excretion of proinflammatory cytokines, which promotes nerve injury and apoptosis (Tator, 1991 #3231). Studies have shown that interleukin (IL)-1β, tumor necrosis factor (TNF)-α, and IL-6 levels are increased in the early stage of SCI [2,3]. The inflammation and apoptosis of the spinal cord are important parts of the mechanism of secondary SCI and affect the recovery and outcome of nerve function after SCI [4,5]. Therefore, the regulatory mechanism of inflammation and apoptosis should be studied. Neuronlike rat adrenal pheochromocytoma cell line (PC-12) originate from neuroblastic cells, which can differentiate into sympathetic-neuron-like cells and thus have been regarded as a tool for the study of molecular processes associated with neuronal differentiation and morphogenesis [6,7]. This cell line is a widely used cell model for studying SCI [8–10]. Lipopolysaccharide (LPS), an endotoxin in the outer membranes of gram-negative bacteria, can trigger the release of a large number of inflammatory cytokines such as TNF-α and IL-6 [11]. Therefore, in this study, we constructed an inflammatory injury model of PC-12 cells using LPS to mimic the second injury of SCI, and cell viability, apoptosis, and release of inflammatory cytokines were measured to evaluate cell injury.

Notoginsenoside R1 (NGR1) is a herbal medicine extracted from panax notoginsenoside, which is well known in the prescription for mediating the micro-circulatory hemostasis in humans [12]. It has been found that the pharmacological properties of panax notoginsenoside are mainly attributed to the effect of NGR1 [13]. NGR1 exhibits potent characteristics of neuroprotective, anti-inflammatory, anti-apoptosis, anti-ischemia-reperfusion injury properties, etc. [14]. NGR1 substantially eliminates oxidative stress, consequently maintains mitochondrial homeostasis, and finally impedes excessive inflammatory responses [15]. Notably, it was demonstrated that panax notoginsenoside produces neuroprotective effects in the rat models of acute spinal cord ischemia-reperfusion injury [16]. NGR1 has an anti-inflammatory effect on SCI injury by controlling miR-132 [17]. However, the exact regulatory mechanisms related to NGR1 in SCI need further investigation.

MicroRNAs (miRNAs) are a subtype of non-coding RNA that is involved in the regulation of a variety of biological processes [18]. In previous studies, miRNAs have been demonstrated to play significant roles in numerous diseases. For instance, miR-182-5p restrains the lymphangiogenesis and tumorigenesis in colon cancer by targeting vascular endothelial growth factor-C (VEGF-C) [19]. MiR-155 silencing attenuates inflammation in chronic obstructive pulmonary disease [20]. MiR-370-3p promotes endothelial cell proliferation and tube formation *via* regulating phosph-small mothers against decapentaplegic 3 (SMAD3) [21]. In addition, numerous miRNAs have been found to be associated with the pathological response after SCI [22]. The dysregulation of miRNAs has shown to cause uncontrolled generation of inflammatory cytokines resulting in various diseases [23]. MiR-301a is located in the first intron of the host gene spindle and kinetochore-associated protein-2 [24]. MiR-301a is described to be implicated in the pathogenesis of inflammation. As widely suggested, miR-301a overexpression exacerbates inflammation [25,26]. Mechanically, studies have found that miR-301a directly regulates cytokines expression or indirectly mediates the transcriptional progress by activating or blunting relevant signaling pathways [25,27]. Moreover, a study indicated that NGR1 mitigates LPS-elicited inflammatory injury in mouse clonal chondrogenic cell line (ATDC5) cells by inhibiting miR-301a [28]. This suggests that NGR1 may interfere with miR-301a under inflammatory conditions. More studies are needed to test whether miR-301a joins in the influence of NGR1 on LPS-stimulated PC-12 cells.

In this study, we further consolidated the protective effect of NGR1 against LPS-triggered inflammatory injury. The molecular mechanisms involved in miR-301a were investigated. We aimed to uncover the association between NGR1 and miR-301a in the pathological response of SCI.

## 2 Materials and methods

### 2.1 Cell culture and treatment

PC-12 (CRL-1721™) and human embryonic kidney (HEK)293T (ACS-4500™) cells were obtained from American Type Culture Collection (ATCC) (Manassas, VA, USA), and cultured in Dulbecco’s Modified Eagle’s medium (DMEM) (cat. no. 10569010; Gibco, Grand Island, NY, USA) containing 10% fetal bovine serum (FBS) (cat. no. SH30070.03; HyClone, Logan, UT, USA) in a humidified 5% CO_2_ condition at 37°C. Inflammatory conditions of PC-12 cells were induced by treatment with doses of LPS (0, 1, 5, and 10 µg/mL; cat. no. L2880; Sigma-Aldrich, MO, USA) for 12 h. NGR1 (0, 10, 20, 30, 40, and 50 µM; cat. no. 80418-24-2; Shanghai Zheyan Biotech Co., Ltd, China) was prepared for 24 h before cells were stimulated by LPS.

### 2.2 Cell transfection

The miR-301a mimics and NC mimics were synthesized by GenePharma (Shanghai, China). The Krüppel-like factor 7 (KLF7) knockdown plasmid vector (sh-KLF7) and sh-NC were constructed by Genearray Biotechnology (Shanghai, China). PC-12 cells (1 × 10^5^ cells/well) were seeded in 6-well plates. Transient transfection with 50 nM oligonucleotides or 2 µg/mL vectors was done utilizing Lipofectamine 3000 (cat. no. L3000-015; Invitrogen, CA, USA). Subsequent analysis was performed after 48 h.

### 2.3 Reverse transcription-quantitative PCR (RT-qPCR)

Total RNA was isolated from the LPS-treated PC-12 cells or spinal cord tissue using TRIzol (cat. no. 15596026; Invitrogen). PrimeScript RT reagent kit (cat. no. HRR037A; Takara, Dalian, China) was used to transcribe RNAs into cDNAs. PCR was performed by using synergy brands (SYBR) Green PCR kit (cat. no. RR086A; TaKaRa) on applied biosystems (ABI) PRISM 7900 Sequence Detector System (Applied Biosystems, Foster City, CA, USA). For the evaluation of the expression of miR-301a, Mir-X™ miRNA First-Strand Synthesis Kit (cat. no. 638313; TaKaRa) was used for reverse transcription. U6 and glyceraldehyde-3-phosphate dehydrogenase (GAPDH) were used as internal controls for miRNA and mRNA expression, respectively. The data calculation was subjected to the 2^−ΔΔCT^ method. The primers were designed by GenePharma and are shown as follows:

miR-301a: 5′-GCCGAGCAGTGCAATAGTATTG-3′ (forward)

5′-CTCAACTGGTGTCGTGGA-3′ (reverse)

KLF7: 5′-ACAAAACAAAAGGGCCACTG-3′ (forward)

5′-GCTGAGAAGTAGCCGGTGTC-3′ (reverse)

GAPDH: 5′-GCAAGTTCAACGGCACAG-3′ (forward)

5′-GCCAGTAGACTCCACGACAT-3′ (reverse)

U6: 5′-CTCGCTTCGGCAGCACA-3′ (forward)

5′-AACGCTTCACGAATTTGCGT-3′ (reverse).

### 2.4 Western blotting

Total protein from the LPS-treated PC-12 cells and spinal cord tissue was extracted using radio-immunoprecipitation assay (RIPA) buffer (cat. no. 9800; Cell Signaling Technology, Inc., USA). Then, the concentration of protein was determined using bicinchoninic acid protein quantitative kit (cat. no. P0010S; Beyotime, Shanghai, China). The protein extractions were separated by 12% sodium dodecyl sulphate-polyacrylamide gel electrophoresis (SDS-PAGE) and transferred on polyvinylidene difluoride (PVDF) membranes (cat. no. IPVH00010; Millipore, Billerica, MA, USA). After blocking with 5% nonfat milk for 1 h, the membranes were incubated with the primary antibodies against Bax (ab32503; 1:1,000, abcam, Cambridge, UK), B-cell lymphoma-2 (Bcl-2) (ab194583; 1:1,000), cleaved-caspase-3 (#9661; 1:1,000, Cell signaling Technology), total-caspase-3 (#14220 S; 1:1,000), IL-6 (ab259341; 1:1,000), TNF-α (ab205587; 1:1,000), IL-1β (ab254360; 1:1,000), KLF7 (ab197690; 1:1,000), β-catenin (ab32572; 1:1,000), cyclin D1 (ab16663; 1:200), c-Myc (ab32072; 1:1,000), and GAPDH (ab181602; 1:10,000) at 4°C overnight. Subsequently, the membranes were incubated with a horseradish peroxidase-conjugated anti-rabbit IgG secondary antibody (cat. no. 7074; Cell Signaling Technology) at room temperature for 2 h. Eventually, an enhanced electrochemiluminescence detection system (Millipore) was applied to visualize the bands.

### 2.5 3-(4,5-Dimethylthiazol-2-yl)-2,5-diphenyltetrazolium bromide (MTT) assay

A total of 3 × 10^3^ PC-12 cells transfected with indicated plasmids were seeded in a 96-well plate for 24 h of incubation, and 0.5% MTT solution (20 µL; cat. no. M1025; Solarbio, Beijing, China) was added. Then, the incubation continued for 4 h at 37°C. Subsequently, dimethyl sulfoxide (cat. no. D2650; Sigma-Aldrich) was added to dissolve the formazan. A microtiter plate reader (Molecular devices, Shanghai, China) was used to record the absorbance at 490 nm.

### 2.6 Flow cytometry analysis

The apoptosis of PC-12 cells was examined using Annexin V fluorescein isothiocyanate (FITC)/propidium iodide (PI) (Annexin V-FITC/PI) apoptosis detection kit (cat. no. A211-01; Nanjing Novizan Biotechnology Co., China). PC-12 cells in 6-well plates at 1 × 10^5^ cells/well were cultured overnight at 37°C. Cells were cultured at 80% confluence and suspended in binding buffer at 1 × 10^6^ cells/mL. Thereafter, 10 µL Annexin V-FITC and PI were added into cells and maintained in the dark for 25 min. Finally, the apoptosis was detected using FACScan (Beckman Coulter, Fullerton, CA) and measured with FlowJo software (Tree Star, CA, USA).

### 2.7 Enzyme linked immunosorbent assay (ELISA)

Spinal cord tissues were mixed with a volume of normal saline 10 times the tissue mass. Then, tissues were placed in a glass homogenizer and were homogenized on ice for 8 min. After centrifugation at 4°C and 5,000 rpm for 5 min, the supernatant was obtained. The supernatants of tissues and PC-12 cells were collected for evaluating the levels of IL-6 (ab234570), TNF-α (ab236712), IL-1β (ab255730) using ELISA kits (Abcam). The absorbance at 450 nm was measured by the use of a microplate reader (Molecular Devices).

### 2.8 Luciferase reporter assay

The online tool (http://www.targetscan.org/vert_70/) was applied to predict putative binding sites between rno-miR-301a and its targets. To test the binding affinity between rno-miR-301a and 3′UTR of KLF7, the wild-type (Wt) or mutated (Mut) 3′UTR of KLF7 was cloned into the pmirGLO reporter vector (cat. no. E1330; Promega, Madison WI, USA) and named as KLF7-Wt and KLF7-Mut. KLF7-Wt/Mut plasmids were co-transfected with miR-301a mimics or NC mimics into HEK293T cells with Lipofectamine 3000. Luciferase activity was detected using the Dual-Luciferase Reporter assay system (Promega) after 48 h.

### 2.9 Animals and NGR1 treatment

Sixty healthy SD rats (30 males and 30 females, weighing 150–180 g) were purchased from Vital River Co. Ltd (Beijing, China). Animals were kept at 24 ± 0.5°C in a comfortable environment with a 12-h light/dark cycle. Each cage had five rats. The rats were divided into four groups: sham, SCI, SCI + NGR1, SCI + NGR1 + XAV939. *N* = 15 each group. All experimental procedures were approved by the Animal Ethics Committee of the First Hospital of Changsha.

In the SCI + NGR1 group, 20 mg/kg NGR1 was intraperitoneally injected daily for the first 2 weeks after SCI. In the SCI + NGR1 + XAV939 group, 0.4 mg/kg XAV939 (cat. no. M1796; Abmole, USA) was intraperitoneally injected daily, and then NGR1 was intraperitoneally injected daily for 2 weeks after SCI.

### 2.10 SCI rat model

The SCI model was established using the modified Allen method [29]. In brief, anesthesia was performed using 10% chloral hydrate (0.33 mg/kg; cat. no. 23100; Sigma-Aldrich) through intraperitoneal injection. The rats were placed in a stereotactic frame. After spinous T 9/10 was removed, the spinal cords were aseptically exposed. Next, a 2 mm diameter striker weighing 10 g was dropped from a height of 25 mm. Neurogenic bladder and hind limb paralysis were induced. The sham rats underwent laminectomy only after anesthesia. After the surgery, the spinal cords were cleaned with saline solution, and the incisions were disinfected with antibiotics for 3 consecutive days to avoid infection. Manual bladder massage was done twice a day to recover bladder function.

### 2.11 Nissl staining

The 20 µm sections were reheated and soaked overnight in a mixed solution of anhydrous ethanol (cat. no. KL809056; KALANG, Shanghai, China) and chloroform (cat. no. 613312-1EA; Sigma-Aldrich) at a 1:1 ratio. The next day, sections were dehydrated in 100% alcohol, 95% alcohol, and distilled water, followed by staining with 0.05% cresyl violet solution (cat. no. GMS80052; GENMED Scientifics Inc., USA) at 40°C for 10 min. The sections were then immediately washed with distilled water, differentiated in 95% ethanol for 10 min, and immersed in xylene (cat. no. 2-10023463; JiangShun Chemical Technology co., China) for 6 min. The sections were observed with an optical microscope after being sealed with neutral gum. The spinal cord tissue lesion and the number of neurons in the anterior horn were assessed from five randomly selected staining sections.

### 2.12 Statistical analysis

Data analyses were processed using GraphPad Prism 7 (GraphPad Inc., USA). The data were shown as the mean ± SD derived from at least three separate experiments. The significant differences between the two groups were analyzed by Student’s *t* test and differences among more than two groups were assessed using a one-way analysis of variance. *p* < 0.05 was considered statistically significant.

## 3 Results

### 3.1 LPS triggers PC-12 cell apoptosis and inflammation

**Figure S1:**
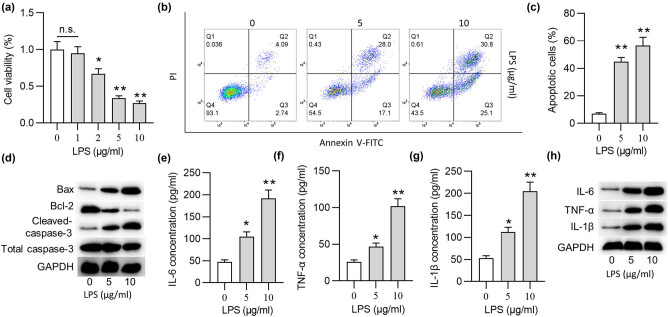
LPS triggers PC-12 cell apoptosis and inflammation. (a) PC-12 cell viability after treatment with LPS for 12 h was detected by MTT. (b and c) PC-12 cell apoptosis after LPS treatment was assessed by flow cytometry. (d) The protein levels of apoptotic genes in PC-12 cells after LPS treatment were measured by western blotting. (e–g) The concentrations of IL-6, TNF-α, IL-1β in PC-12 cells after LPS treatment were assessed by ELISA. (h) The cytokine protein levels in PC-12 cells after LPS treatment were measured by western blotting. *p < 0.05, **p < 0.01.

PC-12 cells were exposed to different concentrations of LPS for 12 h. The viability was detected by MTT. The results showed that the viability of PC-12 cells was significantly decreased after the addition of LPS from 2 µg/mL to 10 µg/mL (*p* < 0.01) (Figure S1a). Flow cytometry analysis was used for investigating the apoptosis of PC-12 cells. As shown, the apoptosis was increased in the presence of 5 and 10 µg/mL LPS (*p* < 0.01) (Figure S1b and c). In addition, the protein levels of Bax and cleaved caspase-3 were elevated while those of Bcl-2 reduced after LPS treatment, as demonstrated by western blotting (Figure S1d). The ELISA results showed that IL-6 (*p* < 0.05; *p* < 0.01), TNF-α (*p* < 0.05; *p* < 0.01) and IL-1β (*p* < 0.05; *p* < 0.01) levels in the culture supernatant of PC-12 cells were increased after LPS was added (Figure S1e–g). Similarly, LPS also upregulated the protein expression of these pro-inflammatory cytokines in PC-12 cells (Figure S1h). Overall, these results indicated that LPS triggered inflammatory damage of PC-12 cells. LPS at the concentration of 5 µg/mL was used for further experiments.

### 3.2 NGR1 reduces the effect of LPS in PC-12 cells

**Figure S2:**
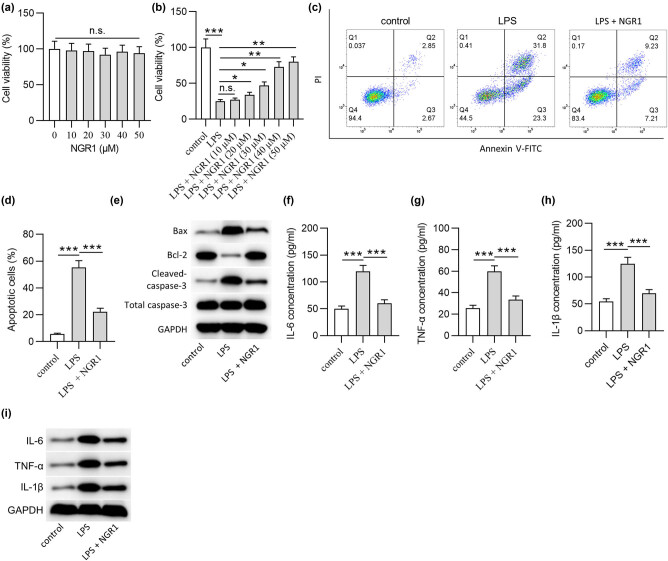
NGR1 reduces the effect of LPS in PC-12 cells. (a) PC-12 cell viability after treatment with NGR1 (10, 20, 30, 40, 50 µM) for 24 h was detected by MTT. (b) The viability of LPS-stimulated PC-12 cells after treatment with different concentrations of NGR1 was detected by MTT. (c and d) The apoptosis of LPS-stimulated PC-12 cells after NGR1 treatment was assessed by flow cytometry. (e) The protein levels of apoptotic genes in LPS-stimulated PC-12 cells after NGR1 treatment were assessed by western blotting. (f–h) The concentrations of IL-6, TNF-α, IL-1β in LPS-treated PC-12 cells after NGR1 treatment were assessed by ELISA. (i) The cytokine protein levels in LPS-treated PC-12 cells after NGR1 treatment were measured by western blotting. *p < 0.05, **p < 0.01, ***p < 0.001.

Next, NGR1 was introduced to determine its role in PC-12 cell damage induced by LPS. The MTT results showed that the treatment with varying doses of NGR1 had no significant influence on the viability of PC-12 cells (Figure S2a), indicating that NGR1 had no significant cytotoxicity on PC-12 cells. However, in the presence of LPS (5 µg/mL), NGR1 (10–50 µM) markedly increased PC-12 cell viability and had the best effect at a concentration of 50 µM (*p* < 0.01) (Figure S2b). Therefore, NGR1 at a concentration of 50 µM was selected for further use. As the flow cytometry results presented, NGR1 reversed the LPS-stimulated increase of PC-12 cell apoptosis (*p* < 0.001) (Figure S2c and d). The elevated protein levels of Bax, cleaved caspase-3, and reduced protein levels of Bcl-2 mediated by LPS were also reversed after NGR1 was introduced (Figure S2e). In addition, in LPS-stimulated PC-12 cells, the levels of IL-6 (*p* < 0.001), TNF-α (*p* < 0.001), and IL-1β (*p* < 0.001) were all reduced in the presence of NGR1, in comparison to the absence of NGR1 (Figure S2f–h). Similar results were observed in the Western blotting analysis, showing that NGR1 decreased the protein levels of these pro-inflammatory cytokines in LPS-treated PC-12 cells (Figure S2i). These data confirmed that NGR1 protected PC-12 cells from LPS-triggered injury.

### 3.3 MiR-301a reverses the effect of NGR1

**Figure 1:**
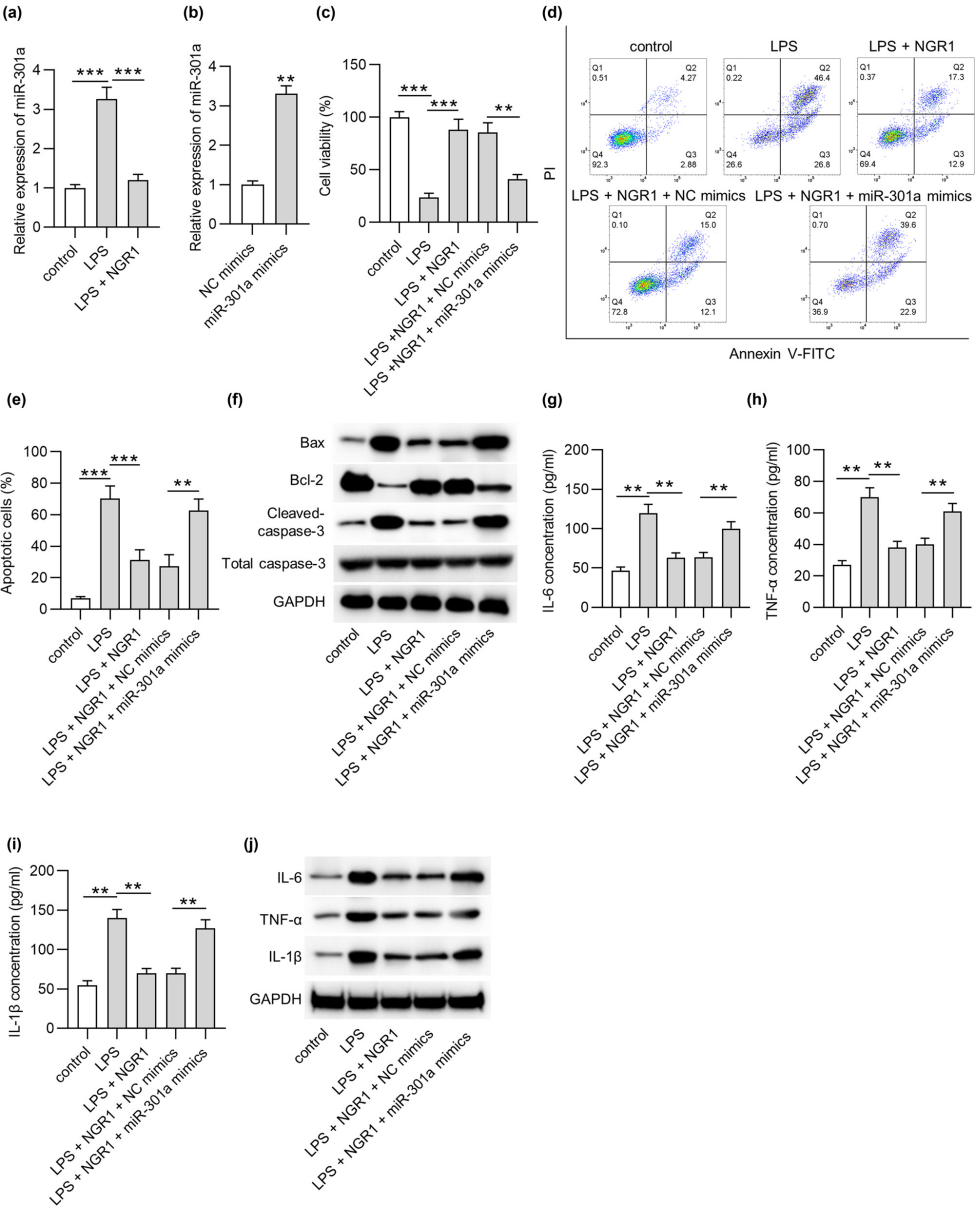
MiR-301a reverses the effect of NGR1. (a) MiR-301a expression in PC-12 cells with LPS treatment or co-treatment of LPS and NGR1 was measured by RT-qPCR. (b) RT-qPCR was used to detect miR-301a overexpression efficiency in PC-12 cells. PC-12 cells were divided into five groups: control, LPS, LPS + NGR1, LPS + NGR1 + NC mimics, and LPS + NGR1 + miR-301a mimics. (c) MTT assay was applied to detect the viability of PC-12 cells in each group. (d and e) Flow cytometry analysis was used to assess the apoptosis of PC-12 cells in each group. (f) The protein levels of Bax, Bcl-2, and cleaved caspase-3 in PC-12 cells were assessed by western blotting. (g–i) The concentrations of IL-6, TNF-α, IL-1β in PC-12 cells were assessed by ELISA. (j) The cytokine protein levels in PC-12 cells were measured by western blotting. **p < 0.01, ***p < 0.001.

Subsequently, the molecular mechanisms addressed by NGR1 were investigated. NGR1 was reported to relieve LPS-elicited inflammatory injury in a miR-301a-silenced manner in ATDC5 cells [28]. Therefore, we investigated whether NGR1 could regulate miR-301a in LPS-stimulated PC-12 cells. As indicated by RT-qPCR, upregulated miR-301a expression was detected in PC-12 cells with LPS treatment (*p* < 0.001), and the miR-301a level was decreased after NGR1 addition (*p* < 0.001) (Figure 1a), implying a relationship between miR-301a and NGR1 in LPS-stimulated PC-12 cells. MiR-301a was effectively upregulated in PC-12 cells after miR-301a mimics were transfected (*p* < 0.01) (Figure 1b). The data from MTT showed that the increased viability of LPS-treated PC-12 cells by NGR1 was reduced after miR-301a overexpression (*p* < 0.01) (Figure 1c). The apoptosis of LPS-treated PC-12 cells was decreased by NGR1 (*p* < 0.01), which was then promoted by miR-301a mimics (*p* < 0.01) (Figure 1d and e). Furthermore, miR-301a overexpression eliminated the effect of NGR1 on Bax, cleaved-caspase-3, and Bcl-2 levels in LPS-treated PC-12 cells (Figure 1f). Furthermore, the levels of IL-6 (*p* < 0.01), TNF-α (*p* < 0.01) and IL-1β (*p* < 0.01) were decreased by NGR1 treatment but restored as a result of miR-301a overexpression (Figure 1g–j). Overall, we concluded that NGR1 might attenuate LPS-triggered PC-12 cell injury by downregulating miR-301a.

### 3.4 NGR1 activates wingless/integrated (Wnt)/β-catenin signaling by upregulating KLF7

**Figure 2:**
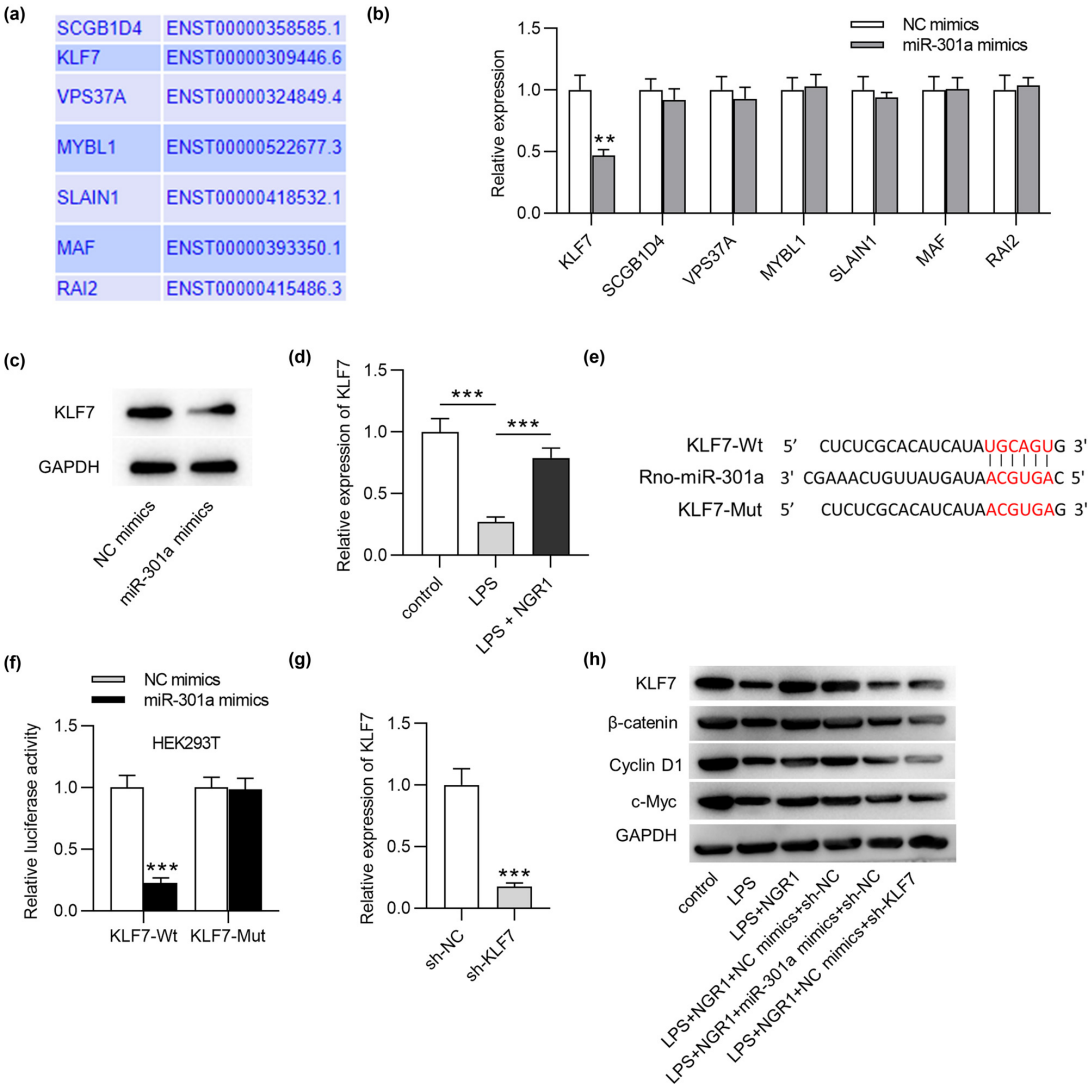
NGR1 activates Wnt/β-catenin by upregulating KLF7. (a) Seven genes ranking ahead in the TargetScan database were found to have a binding site for miR-301a. (b) RT-qPCR analysis was used to measure gene expression after miR-301a overexpression. (c) Western blotting was used to measure KLF7 protein expression after miR-301a overexpression. (d) RT-qPCR was used to measure KLF7 expression in PC-12 cells with LPS treatment or co-treatment of LPS and NGR1. (e) The rno-miR-301a binding site at position 847-853 of KLF7 3′UTR. (f) Luciferase reporter assay was performed in HEK293T cells transfected with KLF7-Wt/Mut reporters and miR-301a mimics or control. (g) RT-qPCR analysis was used to examine KLF7 silencing efficiency. (h) Western blotting was used to measure KLF7 and Wnt/β-catenin-associated gene protein levels in PC-12 cells in the control, LPS, LPS + NGR1, LPS + NGR1 + NC mimics + sh-NC, LPS + NGR1 + miR-301a mimics + sh-NC, and LPS + NGR1 + NC mimics + sh-KLF7 groups. **p < 0.01, ***p < 0.001.

We examined the online tools to identify the targets of rno-miR-301a. Seven genes ranking ahead in the TargetScan database were found to have a binding site for miR-301a (Figure 2a), in which KLF7 mRNA expression was significantly downregulated in miR-301a-overexpressed PC-12 cells (*p* < 0.01) (Figure 2b). The other targets had no significant change. Western blotting showed reduced protein expression of KLF7 after miR-301a overexpression (Figure 2c). In addition, downregulated KLF7 expression was detected in PC-12 cells with LPS treatment (*p* < 0.001), and the KLF7 level was elevated by NGR1 (*p* < 0.001) (Figure 2d). The rno-miR-301a binding site at position 847-853 of KLF7 3′UTR was shown in Figure 2e. Wt or Mut KLF7 sequence was synthesized, and the luciferase activity in HEK293T cells transfected with KLF7-Wt reporters was notably reduced (*p* < 0.001); however, cells transfected with KLF7-Mut reporters had no change in the luciferase activity (Figure 2f). This demonstrated that KLF7 was targeted by miR-301a. Wnt/β-catenin signaling participates in the regulation of apoptosis and inflammatory response after SCI. We then investigated the status of Wnt/β-catenin signaling in LPS-treated PC-12 cells. KLF7 was effectively silenced in PC-12 cells after sh-KLF7 was transfected (*p* < 0.001) (Figure 2g). The results in Figure 2h showed that, in the presence of LPS, KLF7, and Wnt/β-catenin signaling-associated genes (β-catenin, cyclin D1, and c-Myc), protein levels were reduced compared to those in control cells. NGR1 significantly reversed these changes. Strikingly, miR-301a overexpression and KLF7 knockdown then decreased these protein levels restored by NGR1. These findings suggested that NGR1 activated Wnt/β-catenin signaling in LPS-treated PC-12 cells by inhibiting miR-301a and upregulating KLF7.

### 3.5 XAV939 reverses the effect of NGR1 in PC-12 cells

**Figure 3:**
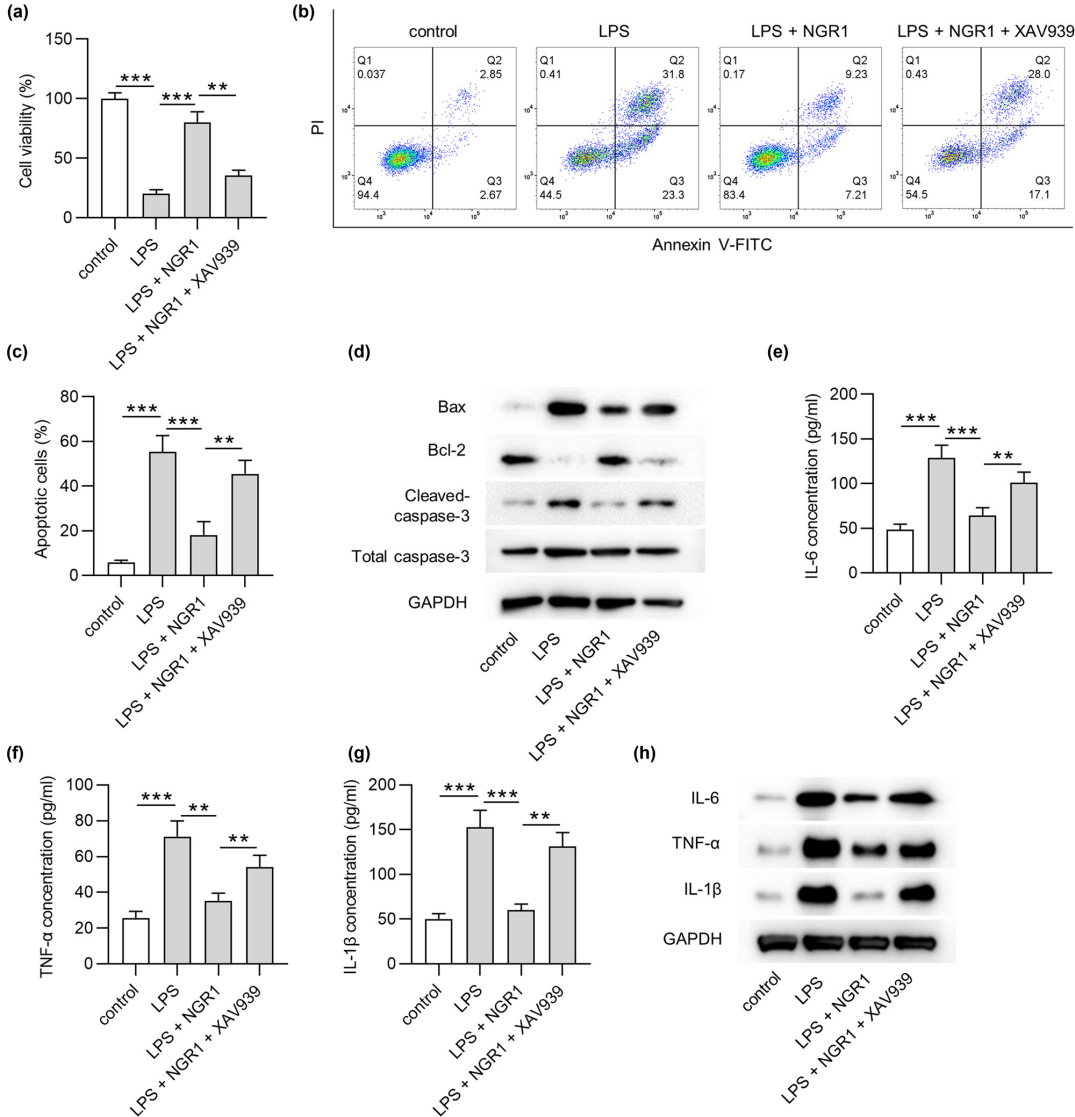
XAV939 reverses the effect of NGR1 in PC-12 cells. PC-12 cells were divided into four groups: control, LPS, LPS + NGR1, and LPS + NGR1 + XAV939. (a) MTT assay was applied to detect the viability of PC-12 cells in each group. (b and c) Flow cytometry analysis was used to assess the apoptosis of PC-12 cells in each group. (d) The protein levels of Bax, Bcl-2, and cleaved caspase-3 in PC-12 cells were assessed by western blotting. (e–g) The concentrations of IL-6, TNF-α, IL-1β in PC-12 cells were assessed by ELISA (Jh). The cytokine protein levels in PC-12 cells were measured by western blotting. **p < 0.01, ***p < 0.001.

We next addressed whether the interfering Wnt/β-catenin pathway could reverse the protective effect of NGR1 against LPS-induced injury in PC-12 cells. We treated PC-12 cells with 50 µM NGR1 either alone or in combination with 10 µM Wnt/β‑catenin inhibitor XAV939 for 24 h. The MTT results showed that the increased viability of LPS-treated PC-12 cells by NGR1 was reduced after the addition of XAV939 (*p* < 0.01) (Figure 3a). The apoptosis of LPS-treated PC-12 cells was decreased by NGR1 (*p* < 0.001), which was elevated by XAV939 (*p* < 0.01) (Figure 3b and c). XAV939 also eliminated the effect of NGR1 on Bax, cleaved-caspase-3, and Bcl-2 levels in LPS-treated PC-12 cells (Figure 3d). Furthermore, XAV939 increased the levels of IL-6 (*p* < 0.01), TNF-α (*p* < 0.01) and IL-1β (*p* < 0.01) in the presence of NGR1 (Figure 3e–h). These data verified that NGR1 inhibited LPS-triggered PC-12 cell injury by activating the Wnt/β-catenin pathway.

### 3.6 NGR1 improves neuronal survival and alleviates inflammation after SCI by activating Wnt/β-catenin signaling

**Figure 4:**
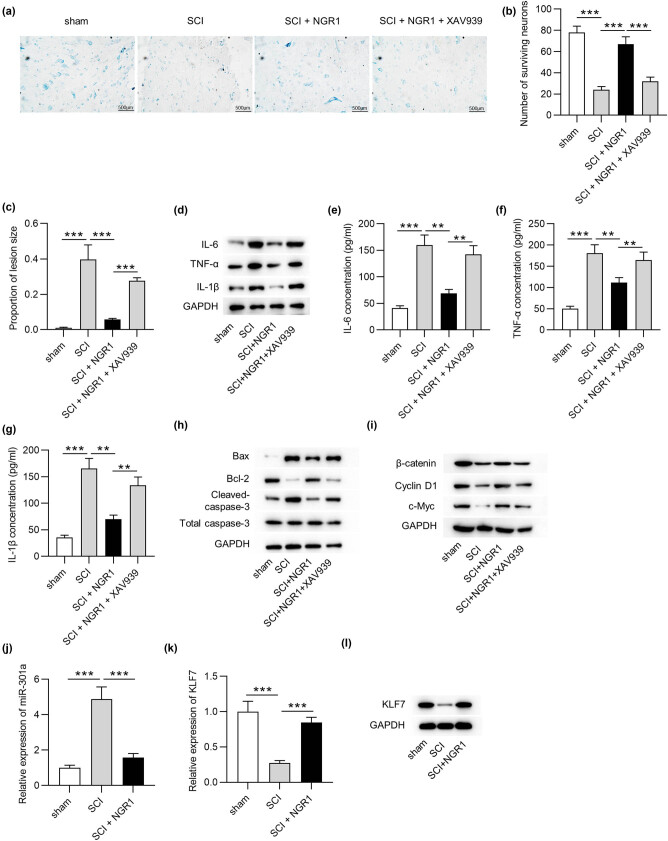
NGR1 improves neuronal survival and alleviates inflammation after SCI by activating Wnt/β-catenin. (a) Nissl staining showing (b) the number of surviving neurons and (c) proportion of lesion size in the spinal cord in the sham, SCI, SCI + NGR1, and SCI + NGR1 + XAV939 groups. (d) Western blotting was used to measure IL-6, TNF-α, IL-1β protein levels in the spinal cord in the sham, SCI, SCI + NGR1, and SCI + NGR1 + XAV939 groups. (e–g) ELISA was used to measure IL-6, TNF-α, IL-1β levels in the spinal cord in the sham, SCI, SCI + NGR1, and SCI + NGR1 + XAV939 groups. (h and i) The protein levels of Bax, Bcl-2, cleaved-caspase-3, β-catenin, cyclin D1, and c-Myc in the spinal cord in the sham, SCI, SCI + NGR1, and SCI + NGR1 + XAV939 groups. RT-qPCR was used to measure (j) miR-301a and (k) KLF7 expression in the spinal cord in the sham, SCI, and SCI + NGR1 groups. (l) KLF7 protein expression in each group. **p < 0.01, ***p < 0.001.

Nissl staining was performed to assess the spinal cord tissue lesion and determine the number of neurons in the anterior horn 14 days after SCI (Figure 4a). The staining results exhibited more neurons in the anterior horn in the NGR1 group than the SCI group (*p* < 0.001), however; pretreatment with XAV939 reduced the number of neurons in the NGR1 group (*p* < 0.001) (Figure 4b). In addition, NGR1 attenuated the proportion of lesion size in the spinal cord of rats after SCI (*p* < 0.001), while XAV939 reversed this effect (*p* < 0.001) (Figure 4c). We further examined the cytokines in the spinal cord of rats in each group. The NGR1 group showed lower levels of IL-6 (*p* < 0.01), TNF-α (*p* < 0.01), and IL-1β (*p* < 0.01) than in the SCI group, which were restored in the NGR1 + XAV939 group (Figure 4d–g). Moreover, NGR1 downregulated Bax and cleaved-caspase-3 protein levels and upregulated Bcl-2 protein levels in SCI rats, while this effect was reversed by XAV939 (Figure 4h). The NGR1 group showed upregulated levels of β-catenin, cyclin D1, and c-Myc compared to the SCI group, which were reduced in the NGR1 + XAV939 group (Figure 4i). In addition, elevated miR-301a expression (*p* < 0.001) and reduced KLF7 expression (*p* < 0.001) were found in rats after SCI (Figure 4j–l). These data indicated that NGR1 promoted the recovery of SCI rats by regulating the miR-301a/KLF7-Wnt/β-catenin pathway.

### 3.7 Schematic diagram of the protective function of NGR1 through the miR-301a/KLF7-Wnt/β-catenin pathway

**Figure 5:**
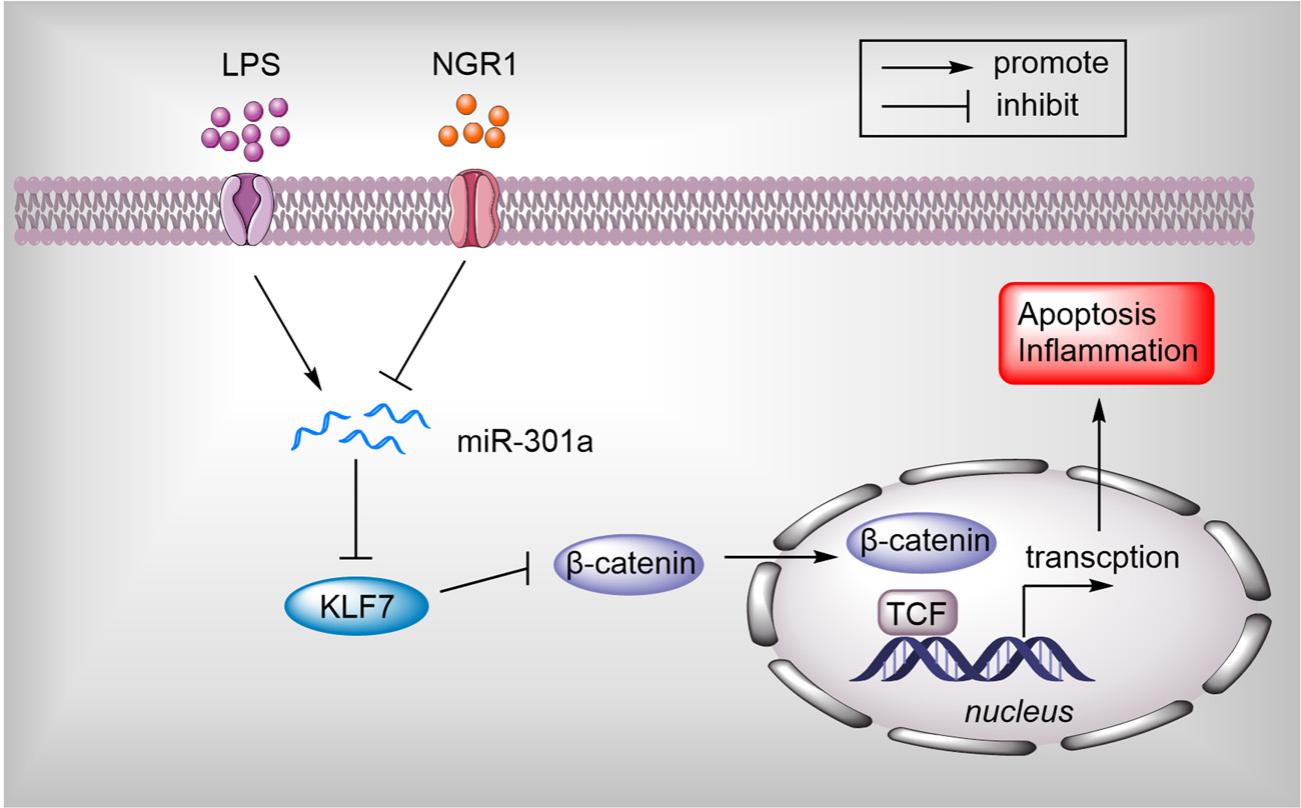
Schematic of the protective function of NGR1 through the miR-301a/KLF7-Wnt/β-catenin pathway.

NGR1-inhibited miR-301a in LPS-stimulated PC-12 cells, subsequently upregulating KLF7 expression and activating the Wnt/β-catenin pathway, leading to the alleviation of apoptosis and inflammation, eventually alleviating the development of SCI (Figure 5).

## 4 Discussion

Increasing studies have demonstrated that SCI is associated with inflammatory response [30], and increased reactive oxygen species activity in SCI leads to an increase in the concentrations of inflammatory cytokines. It has been reported that NGR1 has extensive pharmacological activities, including anti-cancer, anti-inflammatory, cardioprotective, and neuroprotective effects [31]. Previous studies indicated that NGR1 protects different types of cell lines against LPS-induced injury [32,33]. Moreover, NGR1 alleviates LPS-triggered inflammatory damage in PC-12 cells *via* elevating miR-132 [28]. Consistent with previous findings, our study revealed that NGR1 alleviated LPS-triggered apoptosis and inflammation of PC-12 cells. More importantly, our study was the first to use animal experiments demonstrating that NGR1 promoted functional and pathological recovery in rats after SCI by improving the number of neurons and attenuating lesion size in the spinal cord of rats after SCI. These findings provide stronger evidence that NGR1 may be effective in promoting the recovery of SCI rats. Although the biological and pharmacological effects of NGR1 from *in vitro* and *in vivo* studies have been reported, clinical trials with NGR1 are also rare, more controlled trials should be conducted in the future. Meanwhile, NGR1 has been approved by the Food and Drug Administration of China to start clinical arthritis prevention and treatment trials [34]. It provides more possibilities for the application of NGR1 in clinical trials of various diseases, including SCI.

Studies about the associations of apoptosis and inflammation with SCI were frequently reported. For instance, miR-124 attenuates the apoptosis of spinal neurons by targeting guanosine 5'-triphosphate cyclohydrolase I (GCH1) [35]. MiR-223-5p downregulation inhibits the inflammation in microglia of rats with SCI [36]. However, whether miR-301a is responsible for apoptosis and inflammation following SCI is still unclear. MiR-301a is frequently highly expressed in response to chronic inflammation and stimulates the production of cytokines [37,38]. Specific inhibition of miR-301a displays a protective effect against inflammatory injury [27]. However, direct inhibition of miR-301a is inadvisable because miR-301a downregulation is related to elevated chronic inflammation and circulation [39]. Instead of silencing miR-301a, NGR1 functionally interferes with miR-301a, which may be significantly advisable compared to an absolute inhibition of miR-301a. As reported, NGR1 mitigates LPS-elicited inflammatory injury in ATDC5 cells by inhibiting miR-301a [28]. In this study, NGR1 relieved LPS-induced PC-12 cell injury by decreasing the expression of miR-301a. In addition, miR-301a was downregulated in the spinal cord or NGR1-treated SCI rats. Our study was the first to reveal the association between NGR1 and miR-301a in the pathological response of SCI, and clarified a new molecular mechanism of the neuroprotective effect of NGR1 on SCI. The biological effect of NGR1 by controlling miR-301a may be investigated and verified in different pathological conditions.

In our work, bioinformatics analysis showed that KLF7, belonging to the KLFs family, has a potential binding site for miR-301a. KLF7 is considered a key modulator of axon outgrowth. It was shown that KLF7 depletion mainly affects gene activities involving axonal growth, olfactory sensory neuron differentiation, cell adhesion, synaptogenesis, and cytoskeletal dynamics [40]. Moreover, KLF7 exerts a positive role in the central nervous system and peripheral nerve injury by contributing to survival and axonal regeneration in injured nerves [41]. These reports suggest that KLF7 could be beneficial to neuronal regeneration. In the present study, the KLF7 level was downregulated by LPS and increased by NGR1 co-treatment. We demonstrated that miR-301a negatively regulated KLF7 expression. Therefore, the miR-301a/KLF7 axis may be a new molecular mechanism in LPS-induced inflammatory injury. In addition, KLF7 was reported to activate the β-catenin pathway by interacting with coiled-coil domain containing 85c (Ccdc85c) [42]. Studies showed that Wnt pathway activation is beneficial to axonal regeneration and functional recovery [43]. In particular, activation of Wnt after SCI promotes neuronal growth and mitigates neuropathic pain [44]. As previously reported, NGR1 enhances human alveolar osteoblast differentiation in an inflammatory microenvironment as well as facilitates the growth of cultured cortical neurons by activating the Wnt/β‑catenin pathway [45]. Here, the NGR1 activated Wnt/β-catenin signaling in LPS-treated PC-12 cells by inhibiting miR-301a and upregulating KLF7. Furthermore, the protective function of NGR1 was prevented by an inhibitor of the Wnt/β‑catenin pathway. These findings showed that the protective role of NGR1 in SCI may be mediated by the Wnt/β‑catenin pathway.

In conclusion, this study demonstrated that NGR1 decreased neural cell apoptosis and inflammation and contributed to functional recovery in rats after SCI by the miR-301a/KLF7 axis to activate Wnt/β‑catenin signaling. This finding provides a new molecular mechanism for how NGR1 promotes neuroprotection, which might provide a potential treatment for SCI. Further investigations should be conducted to elucidate the role of NGR1 in other neural cell types after SCI and possible molecular mechanisms addressed by NGR1. The therapeutic mechanism of NGR1 in patients with SCI will be studied in a clinic in the future.

## Abbreviations

SCI: spinal cord injury
NGR1: notoginsenoside R1
LPS: lipopolysaccharide
miRNAs: microRNAs
