# Expression of concern “Notoginsenoside R1 alleviates spinal cord injury through the miR-301a/KLF7 axis to activate Wnt/β-catenin pathway”

**DOI:** 10.1515/med-2025-9997

**Published:** 2025-04-01

**Authors:** Editors of Open Medicine

Expression of Concern: Tang Z, Yang C, He Z, Deng Z, Li X. Notoginsenoside R1 alleviates spinal cord injury through the miR-301a/KLF7 axis to activate Wnt/β-catenin pathway. Open Medicine. 2022;17(1):741–755. www.degruyter.com/document/doi/10.1515/med-2022-0461 by Editors of Open Medicine.

With this notice, the Editorial Board of Open Medicine would like to inform readers that Figure 3b was included in the aforementioned article by mistake. This incorrect figure raises questions about the integrity and rigor of the published research. Despite multiple attempts to contact the authors for clarification, no satisfactory response has been received.

The journal’s Editorial Board and the Publication Ethics Committee are investigating the article in accordance with the Committee on Publication Ethics Guidelines and De Gruyter Brill procedures. The Expression of Concern will be updated as soon as the investigation is complete.

